# Association between Stroke History and Clinical Events in Atrial Fibrillation Patients after Valve Replacement

**DOI:** 10.31083/RCM26992

**Published:** 2025-04-23

**Authors:** Xinsheng Yan, Shuwen Lian, Dong Wang, Bao Yan, Litao Zhang, Zhenlu Zhang

**Affiliations:** ^1^Department of Clinical Laboratory, Wuhan Asia General Hospital Affiliated to Wuhan University of Science and Technology, 430056 Wuhan, Hubei, China; ^2^Department of Clinical Laboratory, Chengdu Aerotropolis Asia Heart Hospital, 610500 Chengdu, Sichuan, China; ^3^Department of Pharmacy, Wuhan Asia General Hospital Affiliated to Wuhan University of Science and Technology, 430056 Wuhan, Hubei, China; ^4^Department of Cardiology, Wuhan Asia General Hospital Affiliated to Wuhan University of Science and Technology, 430056 Wuhan, Hubei, China; ^5^Department of Clinical Laboratory, Wuhan Asia Heart Hospital Affiliated to Wuhan University, 430022 Wuhan, Hubei, China

**Keywords:** atrial fibrillation, history of stroke, prognosis, mitral valve replacement, valvular heart disease

## Abstract

**Background:**

The association between stroke history and clinical events after valve replacement in patients with atrial fibrillation (AF) combined with valvular heart disease (VHD) is unclear. Thus, we sought to investigate the relationship between stroke history and clinical events in patients with AF after valve replacement.

**Methods:**

This retrospective cohort study enrolled 746 patients with AF who underwent valve replacement between January 2018 and December 2019 at the Wuhan Asia Heart Hospital. Patient information was collected from the hospital’s electronic medical record system. Patients were categorized based on their stroke history and followed through outpatient visits or by telephone until the occurrence of an endpoint event; the maximum follow-up period was 24 months. Endpoint events included thrombotic events, bleeding, and all-cause mortality. The frequency of thrombotic, hemorrhagic, and fatal events during the follow-up period was compared between the two groups. Independent risk factors for endpoint events were analyzed using multifactorial Cox regression.

**Results:**

The analysis included 746 patients. Over a 24-month follow-up period, there were more total adverse events (hazard ratio (HR) = 2.08, 95% confidence interval (CI) 1.06–4.08, *p* = 0.018), thrombotic events (HR = 10.28, 95% CI 2.85–37.11, *p* < 0.001), and increased all-cause mortality (HR = 5.74, 95% CI 1.84–17.93, *p* < 0.001) in the stroke history group than in the non-stroke history group. Fewer bleeding events were observed in the group with a history of stroke (HR = 0.87, 95% CI 0.37–2.04, *p* = 0.757). A multifactorial Cox regression analysis revealed that a personal history of stroke was an independent risk factor for total adverse events, thrombotic events, and all-cause mortality.

**Conclusions:**

Previous stroke history is significantly associated with adverse events in AF patients following valve replacement.

## 1. Introduction

Valvular heart disease (VHD) is one of the most common clinical heart diseases [1]. Atrial fibrillation (AF) and VHD often occur simultaneously and independently increase the risk of stroke, thromboembolism, and mortality [2, 3]. Meanwhile, heart valve replacement (HVR) remains the primary treatment for VHD [4]. The prognosis of patients can vary significantly depending on the surgery, type of valve replacement, and the combination of cardiovascular risk factors such as hypertension, diabetes mellitus, and stroke.

Patients with a history of stroke are often more difficult to manage and have a poor prognosis [5]. Previous studies have focused on patients with non-valvular atrial fibrillation [6]. The CHA2DS2-VASc score, which incorporates a history of stroke, is widely used to assess embolic stroke risk [7]. The current management of patients with VHD combined with AF is largely based on the overall risk of the patient; nonetheless, assessment of individual risk remains challenging. Moreover, research on other prognostic factors for mitral valve replacement (MVR) in this population remains limited. Therefore, evaluating risk prediction may be of clinical importance and help improve the treatment and management of these patients. Hence, we sought to investigate the impact of stroke history on the occurrence of adverse events following MVR and provide insights for better prognostic management.

## 2. Methods

### 2.1 Study Subjects 

This study included 746 patients with comorbid AF who underwent MVR at the Wuhan Asia Heart Hospital between January 2018 and December 2019. Inclusion criteria included patients diagnosed with AF with concomitant VHD undergoing MVR. Exclusion criteria included non-valvular AF, malignant tumors, septic shock, other serious diseases, or incomplete medical records. Based on previously reported incidences of bleeding and thrombosis [8] and the results of our study, we determined the sample size of the survey using MedCalc 16.2.1 software (MedCalc Software Ltd, Ostend, Belgium). According to our calculations, a study with a two-sided test, a significance of 0.05, a sample size ratio between the stroke history group and the non-stroke history group of 1:2, and a power of 80% would require a sample of 570 cases. This study adhered to the Declaration of Helsinki and was approved by the Ethics Committee of Wuhan Asia Heart Hospital (2023-B017).

### 2.2 Study Design

We investigated a history of previous strokes in these patients using the hospital discharge diagnoses in their medical history. Subsequently, patients were divided into a stroke history group and a non-stroke history group according to the patient’s history of stroke. The stroke history group included all patients who had a medical history of stroke, including transient ischemic attack (TIA). Patients’ baseline clinical data were collected from the electronic medical record system, including sex, age, body mass index (BMI), hypertension, diabetes mellitus, renal insufficiency, cardiac insufficiency, coronary heart disease (CHD), smoking, valve type, and antiplatelet drugs. The results of relevant laboratory tests were also recorded, such as D-dimer, N-terminal pro-brain natriuretic peptide (NT-proBNP), C-reactive protein (CRP), and prothrombin time international normalized ratio (PT-INR).

#### 2.2.1 MVR Procedure

Valve replacement intervention strategies, choice of prosthetic valve type (bioprosthetic/mechanical), and perioperative management strategies were performed according to the 2017 American Heart Association (AHA) guidelines [9]. The surgical procedure included preoperative evaluation, general anesthesia, establishment of extracorporeal circulation, cardiac arrest, removal of the diseased valve, implantation of a prosthetic valve, withdrawal from extracorporeal circulation, and closure of the chest cavity. Anticoagulation was required for at least 3–6 months after surgery for biological heart valves and life after surgery for mechanical valves.

#### 2.2.2 Definition and Outcomes

The major study endpoints were all-cause mortality and the occurrence of thrombotic and bleeding events. The thrombotic events included stroke, systemic embolism, and myocardial infarction. The bleeding events included cerebral hemorrhage, gastrointestinal bleeding, urinary tract bleeding, airway hemorrhage, and bleeding gums. The major bleeding events involved hemorrhages that were intracranial and retroperitoneal. Fatal bleeding was defined as a fall in hemoglobin of at least 20 g/L or the transfusion of two or more units of whole blood or red cells [10]. Minor bleeding was defined as clinically observed bleeding that does not meet the criteria for major bleeding.

#### 2.2.3 Follow up

Clinical follow-up at 3 months, 12 months, and 24 months after HVR was performed. Outpatient or telephone follow-up was used to record and compare the incidence of thrombosis, bleeding, and all-cause mortality between the two groups during the follow-up period.

### 2.3 Statistical Analysis 

Continuous variables with a normal distribution were expressed as the mean and standard deviation (mean ± SD), and the *t*-test was employed for intergroup comparisons. Continuous variables with a non-normal distribution were expressed as the median and interquartile range (IQR), and the Mann–Whitney test was used for intergroup comparisons. Categorical variables were described as frequency and percentage (n (%)), and intergroup comparisons were performed using the Pearson chi-square test or Fisher’s exact test. A comparison of the Kaplan–Meier estimate curves for endpoint events was performed using the log-rank test. Adjusted analyses for the outcomes were performed using Cox regression models to estimate hazard ratios (HRs) with corresponding two-sided 95% confidence intervals (CIs). These confounders were age, sex, BMI, PT-INR, CHD, antiplatelet drugs, valve type, hypertension, and diabetes mellitus. The proportional hazards assumption was checked using the log negative-log survival curve plots for negative logarithms. The statistical significance level was defined as *p* < 0.05. Statistical processing was performed using MedCalc for statistical analysis.

## 3. Results

### 3.1 Clinical Baseline Characteristics 

Four cases had incomplete medical records, resulting in the inclusion of 746 cases. There were 229 patients in the stroke history group and 517 patients in the non-stroke history group. A comparison of the general data between the two groups revealed statistically significant differences in age, hypertension, CHD, and antiplatelet drugs, as shown in Table 1.

**Table 1.** **Baseline patient characteristics**.

Characteristics	Non-stroke history (*n = *517)	Stroke history (*n = *229)	*p-*value
Age (y)	55 (49, 62)	58 (53, 64)	<0.001
Women	350 (67.7)	147 (64.2)	0.349
BMI (kg/m^2^)	21.7 (19.5, 24.2)	21.8 (19.5, 24.2)	0.786
Hypertension	66 (12.8)	59 (25.5)	<0.001
Diabetes mellitus	43 (8.3)	29 (12.7)	0.064
Renal insufficiency	27 (5.2)	19 (8.3)	0.108
Cardiac insufficiency	478 (92.5)	215 (93.9)	0.484
CHD	364 (70.4)	188 (82.1)	<0.001
Smoking	112 (21.7)	57 (24.9)	0.332
Mechanical valves	384 (74.3)	160 (69.9)	0.212
NT-proBNP (pg/mL)	1543 (877, 2594)	1396 (832, 2551)	0.319
CRP (mg/L)	1.71 (0.68, 4.68)	1.58 (0.75, 4.93)	0.852
D-dimer (µg/mL)	1.77 (1.05, 4.04)	1.77 (1.15, 4.22)	0.328
PT-INR	2.16 (1.79, 2.61)	2.14 (1.74, 2.67)	0.741
Antiplatelet drugs	71 (13.7)	46 (20.1)	0.028

Values are expressed as n (%) or median (IQR). y, years; BMI, body mass index; CHD, coronary heart disease; NT-proBNP, N-terminal pro-brain natriuretic peptide; CRP, C-reactive protein; PT, prothrombin time; INR, international normalized ratio; IQR, interquartile range.

### 3.2 Adverse Events 

During the 24-month follow-up period, 11 thrombotic events, 25 bleeding events, and 14 all-cause mortality events were recorded. The group with a history of stroke had nine thrombotic events, seven bleeding events, and 10 all-cause mortality events. In the group without a history of stroke, there were two thrombotic events, 18 bleeding events, and four all-cause mortality events. See Table 2.

**Table 2.** **Comparison of endpoint events in patients with and without a history of stroke (n (%))**.

Events			Non-stroke history (*n = *517)	Stroke history (*n = *229)	Total (*n = *746)
Thrombotic events			2 (0.4)	9 (3.9)	11 (1.5)
	Ischemic stroke		2 (0.4)	2 (0.9)	
	Systemic embolism		0 (0.0)	2 (0.9)	
	Myocardial infarction		0 (0.0)	5 (2.2)	
Bleeding events			18 (3.5)	7 (3.1)	25 (3.4)
	Major bleeding		6 (1.2)	3 (1.3)	9 (1.2)
		Cerebral hemorrhage	5 (1.0)	3 (1.3)	
		Hb decrease 20 g/L or more	1 (0.2)	0 (0.0)	
	Minor bleeding		12 (2.3)	4 (1.7)	16 (2.1)
		Gastrointestinal bleeding	4 (0.8)	2 (0.9)	
		Urinary tract bleeding	4 (0.8)	2 (0.9)	
		Airway hemorrhage	3 (0.6)	0 (0.0)	
		Bleeding gums	1 (0.2)	0 (0.0)	
All-cause mortality			4 (0.8)	10 (4.4)	14 (1.9)
	Cardiac death		1 (0.2)	7 (3.1)	
	Death from lung infection		0 (0.0)	1 (0.4)	
	Stroke death		2 (0.4)	1 (0.4)	
	Unexplained deaths		1 (0.2)	1 (0.4)	
Total			24 (4.6)	26 (11.4)	50 (6.7)

Hb, hemoglobin.

**Fig. 1.**
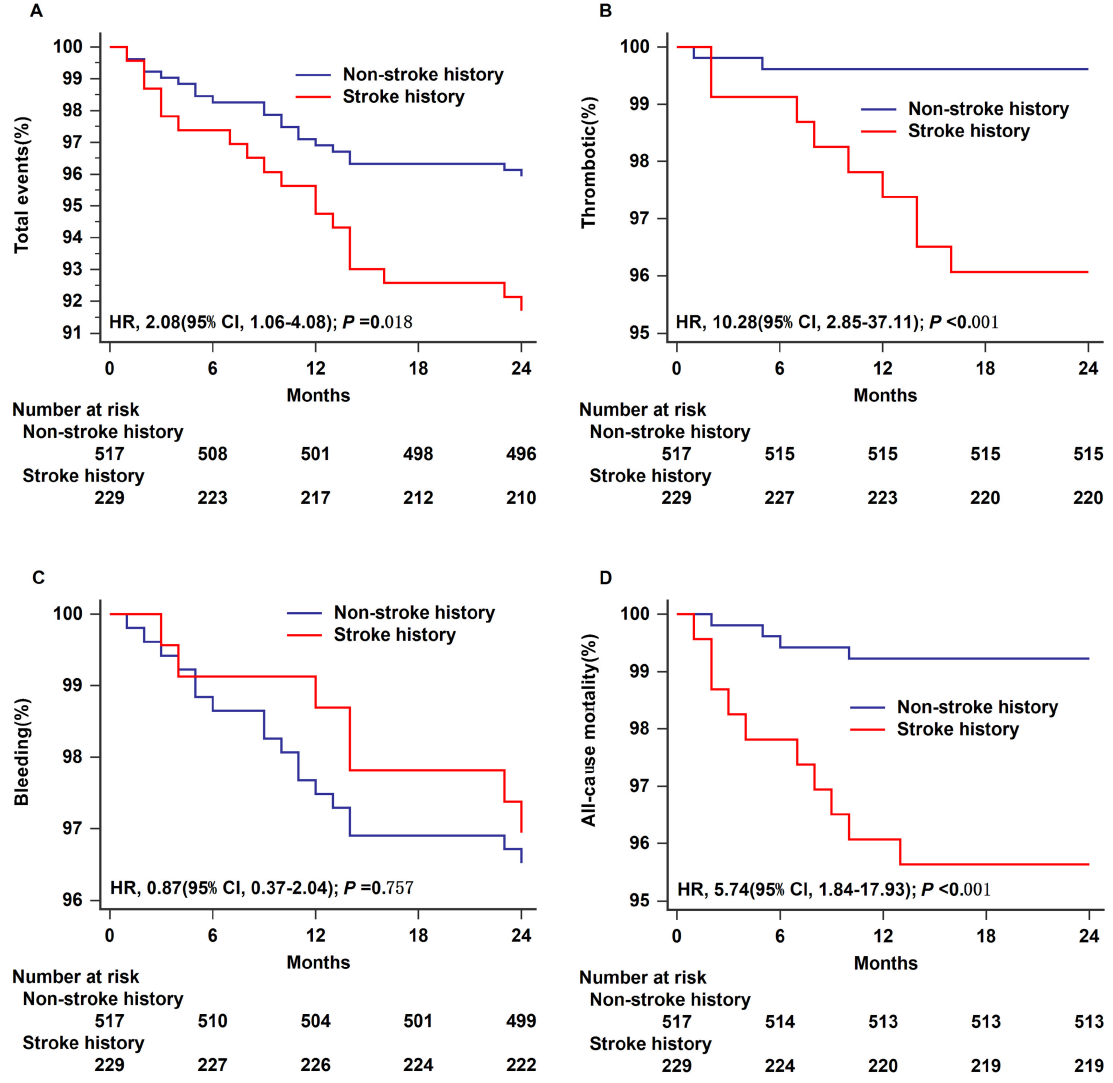
**Time-to-event analysis for outcomes in different groups within 24 months**. Total events (A), thrombotic (B), bleeding (C), and all-cause mortality (D) are shown separately. HR, hazard ratio; 95% CI, 95% confidence interval.

The Kaplan–Meier survival curves for each type of postoperative endpoint event in the two groups are shown in Fig. 1. The log-rank test was used to compare the Kaplan–Meier estimate curves for total events, thrombotic events, bleeding events, and all-cause mortality in the analysis. Compared with the non-stroke history group, the stroke history group experienced a higher incidence of total adverse events (HR = 2.08, 95% CI 1.06–4.08, log-rank *p* = 0.018; Fig. 1A), a greater number of thrombotic events (HR = 10.28, 95% CI 2.85–37.11, log-rank *p* < 0.001; Fig. 1B), and increased all-cause mortality (HR = 5.74, 95% CI 1.84–17.93, log-rank *p* < 0.001; Fig. 1D). However, fewer bleeding events were observed in the stroke history group (HR = 0.87, 95% CI 0.37–2.04, log-rank *p* = 0.757; Fig. 1C).

### 3.3 Analysis of Factors Influencing the Occurrence of Adverse Events in Patients

Multifactorial Cox regression analysis showed that after controlling for age, sex, BMI, PT-INR, CHD, antiplatelet drugs, valve type, hypertension, and diabetes mellitus, a previous history of stroke was still associated with higher total clinical events (HR = 1.99, 95% CI 1.05–3.78, *p* = 0.036), thrombotic events (HR = 8.83, 95% CI 2.17–46.46, *p* = 0.006), and all-cause mortality (HR = 4.72, 95% CI 1.43–15.53, *p* = 0.011), whereas there was no correlation with bleeding events (HR = 0.87, 95% CI 0.35–2.15, *p* = 0.762; Table 3). Log negative-log survival curve plots were used to test the assumption of equal proportional hazards (PH), which showed that the basic assumption was correct. The Cox regression model was appropriate for the study data.

**Table 3.** **Multifactorial Cox regression analysis of the relationship between personal history of stroke and various endpoint events**.

Events	Unadjusted			Adjusted^*^		
	HR	95% CI	*p*-value	HR	95% CI	*p*-value
Total events	2.08	1.06–4.08	0.018	1.99	1.05–3.78	0.036
Thrombotic events	10.28	2.85–37.11	<0.001	8.83	2.17–46.46	0.006
Bleeding events	0.87	0.37–2.04	0.757	0.87	0.35–2.15	0.762
All-cause mortality	5.74	1.84–17.93	<0.001	4.72	1.43–15.53	0.011

^*^Adjusted for age, sex, BMI, PT-INR, CHD, antiplatelet drugs, valve type, hypertension, and diabetes mellitus. HR, hazard ratio; 95% CI, 95% confidence interval; BMI, body mass index; PT-INR, prothrombin time international normalized ratio; CHD, coronary heart disease.

## 4. Discussion

Although MVR is a common and safe procedure, there remains a high risk of postoperative thrombosis and bleeding-related complications, which can negatively impact patient survival [11]. Thus, identifying predictors of long-term mortality and associated adverse events is crucial. Patients with VHD typically have worse outcomes, such as stroke, systemic embolism, major bleeding, or all-cause mortality, compared to non-VHD patients [12]. This study examined the incidence of adverse events two years after MVR in patients with AF and VHD, focusing on the impact of a history of a previous stroke. Our results indicate that the occurrence of thrombotic events and all-cause mortality was significantly higher in the group with a history of stroke, highlighting stroke history as an independent risk factor for postoperative adverse events in patients with AF and VHD.

Rost* et al*. [13] studied 5973 (28.3%) patients with a prior history of ischemic stroke (IS) or TIA compared with 15,132 patients without prior IS/TIA. Those patients with prior IS/TIA exhibited an elevated risk of thromboembolism and hemorrhage. Demirel *et al*. [14] evaluated the incidence of a previous stroke and its impact on prognosis in 958 transcatheter aortic valve implantation (TAVI) patients with a maximum follow-up of 5 years and found that an earlier stroke was significantly associated with all-cause mortality. These studies support the findings of the present study. Furthermore, stroke has a high recurrence rate, and Bando *et al*. [15] demonstrated that a history of stroke after mechanical MVR was 2.57 times more likely to occur in patients with a stroke than those with no stroke history.

Previous research has demonstrated that stroke is a complex condition with various risk factors, including age, gender, ethnicity, hypertension, hyperlipidemia, diabetes mellitus, cardiac disease, smoking, alcohol consumption, BMI, diet, exercise, and genetics [16]. In this current study, individuals with a prior history of stroke were typically older and had more underlying health conditions, including cardiovascular disease, diabetes mellitus, and hypertension, which significantly differed from those without a history of stroke. This suggests that stroke may contribute to a poorer prognosis. Among the risk factors mentioned, age, sex, race, and genetics are non-modifiable. A study on factors influencing survival post-MVR for ischemic heart disease found that one-year mortality was linked to a history of coronary artery bypass graft (CABG) surgery and age. In contrast, mortality after one year was associated with diabetes mellitus, renal insufficiency, and age [17]. Additionally, research by Czer *et al*. [18] on patients undergoing MVR revealed that the presence of coronary artery disease decreased long-term survival, even after adjusting for age, sex, left ventricular ejection fraction, and valvular disease. Sankaramangalam *et al*. [19] also reported on a meta-analysis of 15 survival studies on patients receiving transcatheter aortic valve replacement (TAVR), showing a higher 1-year mortality rate in those with combined coronary artery disease compared to those without (HR = 1.21, 95% CI 1.07–1.36). Genetic factors, such as parental and family history, are also recognized as non-modifiable risk factors for stroke, increasing the overall risk of experiencing a stroke [20]. Several genes, including *PITX2*, *ZFHX3*, *HDAC9* [21, 22], *FOXF2* [23], *GUCY1A3* [24], and *GCH1* [25], have been identified to increase the risk of developing ischaemic stroke. Up to one-third of patients may develop AF within 3 months after valve replacement [26], as both AF and stroke share many risk factors [27]. Research has shown that individuals with AF have a higher risk of stroke and mortality, regardless of the sequence in which AF and stroke occur [28]. Furthermore, patients who develop AF after TAVR have significantly increased risks of mortality, stroke, and bleeding compared to those who do not develop AF. Studies have also confirmed that hypertension and diabetes mellitus are important predictors of mortality after valve surgery [17, 29]. In addition, stroke increases the risk and severity of cognitive impairment [30] as well as post-stroke depression, which complicates postoperative care and has been linked to an increased mortality rate, diminished recovery, more pronounced cognitive deficits, and a reduction in quality of life [31].

Therefore, we have the potential to help reduce adverse events through effective interventional treatment of modifiable factors such as hypertension, hyperlipidemia, diabetes mellitus, smoking, alcohol consumption, BMI, diet, and exercise. A meta-analysis involving 33,774 patients with either ischemic stroke or TIA across eight studies revealed that the use of antihypertensive medications in these patients resulted in a 1.9% reduction in the incidence of stroke [32]. Stroke is treated using numerous therapeutic strategies, including medications, cellular therapy, non-invasive brain stimulation, telerehabilitation, and therapies that target specific symptoms (e.g., hemiparesis, speech disorders, and depression). Since immune-mediated inflammatory responses persist after stroke, studies have found that regulatory T cells (Tregs) have a role in limiting immune and inflammatory responses; thus, Tregs hold promise as an immunotherapy option for stroke treatment [33]. Cell therapy, particularly with mesenchymal stem cells (MSCs), can potentially protect white matter and promote functional recovery [34]. Non-invasive brain stimulation, such as transcranial direct current stimulation (tDCS) and repetitive transcranial magnetic stimulation (rTMS), has shown positive results in improving cognitive and language function [35]. Given the complex pathophysiology of stroke and the different risk factors that accompany it, we need to consider potential therapeutic strategies that target these mechanisms for more personalized and precise healthcare. Therefore, more research is required to optimize treatment dosage, duration, and therapies for specific patient groups [36]. Postoperative treatment strategies for MVR may be superimposed on stroke risk factors. Careful investigation of the history of stroke, better patient education, and more frequent follow-up of these patients, in addition to relevant predictive models or scoring systems, may help manage patients after MVR.

Indeed, a previous study confirmed that adding an antiplatelet agent reduced the risk of thromboembolic events and total mortality compared to using anticoagulation alone and that antiplatelet therapy increased the risk of major hemorrhage [37]. This is inconsistent with the results of the present study, although this may be because antiplatelet therapy after MVR is only used when there are other indications for antiplatelet therapy [4]. Meanwhile, the routine addition of aspirin to vitamin K antagonists (VKA) therapy remains a topic of debate [38]. In the stroke history group of the present study, even though a higher percentage of patients were using antiplatelet agents (20.1% vs. 13.7%), there were also more cases of comorbid coronary artery disease [39]. Asian physicians are generally cautious about the use of anticoagulants because of the risk of bleeding in this population [40]. These factors may have counteracted the effect brought about by the use of antiplatelet agents.

There are several limitations to consider in this study. First, it was conducted at a single center, which may have introduced selection bias. Second, as a retrospective study, intraoperative information was not sufficiently obtained due to data limitations. We observed that postoperative adverse events were mainly concentrated in the first 12 months, which may also be related to perioperative factors, and further studies are needed. Third, this study examined patients with valvular heart disease, and whether the conclusions of this study can be extended to the non-valvular population needs to be confirmed by further research due to significant differences in pathogenesis, management strategies, and treatment modalities.

## 5. Conclusions

The clinical risk factor of stroke history was significantly associated with higher rates of mortality and thrombotic events after MVR. Therefore, identifying this risk factor may be useful in risk-stratifying patients with AF undergoing MVR and developing appropriate anticoagulation strategies.

## Availability of Data and Materials

The datasets used and/or analyzed during the current study are available from the corresponding author on reasonable request.
